# Effects and costs of home-based training with telemonitoring guidance in low to moderate risk patients entering cardiac rehabilitation: The FIT@Home study

**DOI:** 10.1186/1471-2261-13-82

**Published:** 2013-10-08

**Authors:** Jos J Kraal, Niels Peek, M Elske van den Akker-Van Marle, Hareld MC Kemps

**Affiliations:** 1 Department of Medical Informatics, Academic Medical Center – University of Amsterdam, Amsterdam, the Netherlands; 2 Department of Medical Decision Making, Leiden University Medical Center, Leiden, the Netherlands; 3 Department of Cardiology, Máxima Medical Center, Veldhoven, the Netherlands

**Keywords:** Cardiac rehabilitation, Home-based training, Telemonitoring, Physical fitness, Physical activity

## Abstract

**Background:**

Physical training has beneficial effects on exercise capacity, quality of life and mortality in patients after a cardiac event or intervention and is therefore a core component of cardiac rehabilitation. However, cardiac rehabilitation uptake is low and effects tend to decrease after the initial rehabilitation period. Home-based training has the potential to increase cardiac rehabilitation uptake, and was shown to be safe and effective in improving short-term exercise capacity. Long-term effects on physical fitness and activity, however, are disappointing. Therefore, we propose a novel strategy using telemonitoring guidance based on objective training data acquired during exercise at home. In this way, we aim to improve self-management skills like self-efficacy and action planning for independent exercise and, consequently, improve long-term effectiveness with respect to physical fitness and physical activity. In addition, we aim to compare costs of this strategy with centre-based cardiac rehabilitation.

**Methods/design:**

This randomized controlled trial compares a 12-week telemonitoring guided home-based training program with a regular, 12-week centre-based training program of equal duration and training intensity in low to moderate risk patients entering cardiac rehabilitation after an acute coronary syndrome or cardiac intervention. The home-based group receives three supervised training sessions before they commence training with a heart rate monitor in their home environment. Participants are instructed to train at 70-85% of their maximal heart rate for 45–60 minutes, twice a week. Patients receive individual coaching by telephone once a week, based on measured heart rate data that are shared through the internet. Primary endpoints are physical fitness and physical activity, assessed at baseline, after 12 weeks and after one year. Physical fitness is expressed as peak oxygen uptake, assessed by symptom limited exercise testing with gas exchange analysis; physical activity is expressed as physical activity energy expenditure, assessed by tri-axial accelerometry and heart rate measurements. Secondary endpoints are training adherence, quality of life, patient satisfaction and cost-effectiveness.

**Discussion:**

This study will increase insight in long-term effectiveness and costs of home-based cardiac rehabilitation with telemonitoring guidance. This strategy is in line with the trend to shift non-complex healthcare services towards patients’ home environments.

**Trial registration:**

Dutch Trial Register: NTR3780. Clinicaltrials.gov register: NCT01732419

## Background

Cardiovascular disease is a major cause of morbidity and mortality accounting for approximately 40,000 annual deaths in the Netherlands [1]. In 2007, associated healthcare costs were estimated at €6.9 billion [2], almost 10% of the total healthcare costs in the Netherlands. It is expected that, due to ageing, this economic burden will increase over the next decades. Cardiac rehabilitation (CR) is a multidisciplinary intervention aiming at physical and psychosocial recovery after an acute coronary syndrome (ACS) or cardiac intervention (coronary revascularization procedure or valve surgery). In 1995 the Dutch CR committee released the first Dutch multidisciplinary CR guideline with updates in 2004 and 2011 [3]. Since this period, CR has become a fully reimbursed treatment in the Netherlands. Since 1990, the number of possible treatment modalities has gradually extended to four group-based interventions (exercise training, education therapy, lifestyle change therapy, and relaxation- and stress management). Exercise training is widely considered as a crucial part of CR [4] and therefore plays an important role in current CR programs. Exercise-based CR has proven to have beneficial effects on morbidity and mortality [5]. Therefore, it is highly recommended in clinical guidelines [3,6,7]. Nowadays, exercise training is reimbursed in the Netherlands only when performed under direct medical supervision in a hospital or specialized CR clinic.

### Barriers in cardiac rehabilitation

Despite proven benefits, availability of comprehensive guidelines, adequate reimbursement and the widespread availability of CR in the Netherlands, CR uptake remains low. A recent study in a large Dutch cohort showed that only 28.5% of eligible patients actually receive CR [8]. In this study, a longer travelling distance to the nearest CR provider was strongly associated with lower CR uptake, suggesting that transport difficulties and/or lack of time form important barriers for patients to take part in centre-based CR programs. Other patient-related factors impeding participation in centre-based CR programs include problems in scheduling due to work resumption, care of dependents, and reluctance to take part in group-based therapy [9]. Besides low uptake of CR, its long-term effectiveness is limited because traditional centre-based exercise training programs are primarily aimed at improving short-term exercise capacity rather than at inducing long-term lifestyle changes [10]. The graduation from a supervised to an unsupervised environment remains a pivotal event that is associated with a loss of effectiveness, often resulting in a decline in physical fitness and activity levels after the initial rehabilitation period [11,12].

### Home-based exercise training

To address the abovementioned problems, there is a need for innovative rehabilitation methods that aim at an increase of CR uptake and more sustained effects on physical fitness and physical activity. As such, home-based exercise training has the potential to improve participation in CR programs [9,13,14], especially in the younger working population and patients with transport difficulties. A meta-analysis showed that home-based exercise training was equally effective as centre-based exercise training in improving short term exercise capacity in CR patients [15]. Studies published afterwards showed similar effects of home-based training in low to moderate risk CR patients [16], in patients with chronic heart failure [13,17], after coronary artery bypass grafting [18,19], and in elderly CR patients (i.e. ≥ 65 years) [20]. Two of these studies [18,19] did not use monitoring during home-based exercise training, while the others employed a variety of monitoring strategies such as telephone guidance [13,17,21] and home visits [20]. In addition, the training protocols show considerable variation with respect to duration, ranging from 8 to 52 weeks, and exercise intensity, ranging from low-level walking [22] to high intensity aerobic training [18]. Despite these variations in study populations, monitoring strategies and training protocols, all mentioned studies showed beneficial effects on short term exercise capacity after home-based training, and moreover, these training programs were equally safe as centre-based exercise training.

Although home-based exercise training can be considered safe and short-term results are promising, long term effects on physical fitness and activity levels remain questionable. Whereas Prescott et al. [23] showed an initial improvement in exercise capacity after eight weeks of home-based exercise training in CHF patients, exercise capacity levels decreased to baseline at one year follow-up. These results are in line with a study by Dracup et al. [22], also showing a decline in exercise capacity in the 6 months following a guided home-based walking program. A possible explanation for these findings may be the sudden translation from a structured training program to independent exercise [12]. To facilitate this transition, the patient’s responsibility for performing exercise training should be triggered at an early stage of the rehabilitation process by the development of self-management skills [24]. Important self-management skills for maintaining behavioural change are self-efficacy, action planning, problem solving and decision making [25]. According to Lorig et al., self-efficacy, the confidence in one’s own abilities to execute and complete a task, is required to maintain behavioural change. When home-based training is initiated in the early stages of CR, patients will develop a familiarity with exercising at home. When coaching and support from a physical therapist is adequate, patients’ confidence in performing independent exercise can be expected to grow during the rehabilitation process. When CR and the supervision of a physical therapist ends, patients should have developed sufficient confidence in their own ability to maintain independent exercise. Other relevant self-management skills are action planning, problem solving and decision making [25]. The early initiation of exercise at home forces the patients to plan their own training schedule. However, problem solving and decision making with respect to training duration and intensity can only be performed adequately when patients receive feedback from health care professionals based on actual training data. Also, patients should be able to monitor their training data themselves to be able to detect and solve problems at an early stage. Therefore, we hypothesize that remote monitoring of patients during home-based CR can be highly effective in improving self-management skills. Supervision of a physical therapist will be important during the first stage of CR, but patients will develop action planning, problem solving and decision making skills to organize, evaluate and adapt their training schedule during CR.

As mentioned above, development of self-management skills can be expected to improve by application of remote patient monitoring that enable health care professionals and patients to review and discuss measured training data during the initial rehabilitation period. With the broad availability of wearable monitoring devices and internet access in most households, ample opportunities exist to create such innovative rehabilitation service. Preliminary studies using remote monitoring of physical activities and online coaching show promising results. Reid et al. [26] reported improved physical activity levels at one year follow up in ACS patients after participation in an internet-based activity program with educational tutorials and remote monitoring of self-reported physical activities. In their study, physical activity levels were assessed by a pedometer and a self-reported leisure-time questionnaire. Similar results were shown in randomized controlled trials in CR patients [27], patients after initial centre-based CR [28], and in patients with peripheral vascular disease [29], all demonstrating improved self-reported physical activity levels after pedo-/accelerometer based interventions with online counselling as compared to usual care and supervised exercise respectively. Follow-up duration of these studies, however, were relatively short (3–6 months).

### Proposed solution

To improve long-term effectiveness of home-based exercise training in CR patients, we propose to combine homebased training with telemonitoring of physical training parameters and online coaching. In this way, we aim to prepare patients more thoroughly for independent exercise without direct medical supervision. Although cost-effectiveness of previous home-based exercise training strategies appeared similar to regular centre-based exercise training [30], costs of this particular intervention using telemonitoring guidance has not been established. We hypothesize that costs of this home-based intervention do not exceed costs of regular CR, while improvements in physical activity and physical fitness can be maintained at the long-term.

### Study objectives

The objective of the present study is to investigate whether home-based exercise training with telemonitoring guidance results in better long-term physical fitness and activity levels than regular centre-based exercise training. Furthermore, both training strategies will be compared with respect to training adherence, patient satisfaction, health-related quality of life, and costs. We hypothesize that home-based training with telemonitoring guidance using objective training data during the rehabilitation period will increase motivation and self-efficacy for independent exercise in CR patients on the long term, resulting in superior increase in physical fitness and physical activity levels. In addition, we expect that the investments in monitoring devices and ICT services are compensated for by lower direct medical costs in the home-based training group due to fewer supervised exercise training sessions.

## Methods/design

### Study design

This study is designed as a monocentre randomized controlled trial at Máxima Medical Centre Veldhoven in the Netherlands. All subjects are requested to provide written informed consent before study entry. Data are collected at baseline (T0), after three months (T1) and after 12 months (T2). Patient recruitment has started in January 2013. The protocol for this study was approved by the Institutional Review Board of the Máxima Medical Centre Veldhoven in the Netherlands. The trial is registered at ClinicalTrials.gov with registration number NCT01732419.

### Study population and randomization

All patients entering outpatient CR after an ACS (myocardial infarction or unstable angina) or a revascularization procedure (percutaneous coronary intervention (PCI) or coronary artery bypass grafting (CABG)) with a low to moderate risk of further events are considered for participation. Patients are classified as low to moderate risk for further events by a cardiologist, based on the following criteria described in the Dutch cardiac rehabilitation practice guideline [31]:

▪ Stable medical condition.

▪ No severe psychological and/or cognitive disorders.

▪ No angina pectoris during exercise.

▪ Left ventricular ejection fraction > 40%.

▪ No cardiac arrhythmias during exercise.

▪ No significant valvular heart disease.

▪ No congenital heart disorders limiting exercise capacity.

▪ No implantable cardioverter-defibrillator (ICD).

▪ No comorbidities that affect rehabilitation (e.g. chronic obstructive pulmonary disease, diabetes mellitus, locomotive disorders).

Additional inclusion criteria include access to internet facilities and PC at home. After baseline measurements, patients are randomly allocated to either home-based or centre-based training. Allocation is based on randomization with variable block size (two or four), performed with dedicated computer software by a researcher (NP) who is not present at the time of allocation. To conceal allocation, numbered and sealed opaque envelopes are opened between the baseline cardiopulmonary exercise test and the start of exercise training. All treatment modalities other than physical training take place at the outpatient clinic as usual (i.e. education, lifestyle change therapy, and/or relaxation and stress management therapy).

### Exercise training program

Exercise training is prescribed according to current recommendations of the European Association of Cardiovascular Prevention and Rehabilitation of the European Society of Cardiology [32]. In both groups patients participate in a 12-week training program with at least two training sessions of 45 to 60 minutes per week. Patients are instructed to exercise with a training intensity of 70-85% of their maximal heart rate, which is assessed during maximal cardiopulmonary exercise testing at baseline.

Patients in the centre-based training (CT) group receive group-based training sessions at the outpatient clinic under direct supervision of two or three physical therapists specialized in CR. Group size varies between 6 to 8 participants and the patients receive an individually tailored training program on a treadmill or an electromagnetically braked cycle ergometer. During the training period, physical therapists will record attendance. An evaluation of the 12-week training period will take place during the last centre-based training session. During this session the physical therapist encourages the patient to continue their physical activities in their own environment.

In the home-based training (HT) group, the first three training sessions are performed at the outpatient clinic under direct supervision of a physical therapist. During these sessions, patients are familiarized with training duration and intensity and they are instructed on how to use the wearable heart rate monitoring device (Garmin Forerunner 70). In addition, patients are asked about their preferred training modality in their home environment (e.g. cycling, walking/running, workout at health club), and given advice on how to implement this. Patients are instructed to wear the heart rate monitoring device during training sessions and to upload the recorded heart rate data to a web application (Garmin Connect) through the internet. Patients can use Garmin Connect to review their training data graphically and to relate these data to their personal goals. Training data are also accessed through Garmin Connect by a personal coach (exercise specialist or physical therapist specialized in CR). The coach provides feedback on training frequency, duration and intensity once a week by telephone. Regarding safety issues when exercising at home, patients are asked to contact either the rehabilitation centre staff or their general practitioner if they experience any symptoms during or after exercising. After 12 weeks, the weekly coaching sessions are terminated. However, the patients are advised to continue their training with the heart rate monitoring device and web application.

### Telemonitoring guidance

After three supervised training sessions in the outpatient clinic, patients in the HT group start training in their home environment. The coach remotely supervises the training sessions performed at home and offers appropriate support via telephone using a semi-structured interview. In the process of coaching the patient, two important factors are identified. First, in order to coach a patient properly, it is essential that goals are set at the start of the rehabilitation together with the patient. This not only improves the rate of success as it sets the locus of control internally [33], but also makes it possible to coach each patient on its own goals and tailor the coaching sessions to the patient in question. Secondly, self-efficacy should be enhanced by letting the patient take responsibility of achieving the goals that has been set. In order to achieve this, the coach will use principles of Motivational Interviewing during the telephone calls. Motivational Interviewing has been developed as method of approaching people that are engaged in behavioural changes and appears effective in various fields [34]. Its main features are client-centred and non-judgmental techniques, without confronting and arguing on themes that concern the content.

The purpose of the supervision by telephone is three-fold. First, it should be checked whether patients are correctly executing the training in their home environment and whether the training schedule or training modality does not lead to injuries or adverse events. Second, it should be checked whether the patients are adherent to the training schedule and making progress in their training goals or whether they experience motivational problems. Third, if non-adherence or motivational problems exist, motivational interviewing principles are used to detect and resolve barriers, activate patients’ motivation and enhance self-efficacy. The training schedule typically consists of a warm-up phase, a core phase and a cooling down phase. Each phase has a predefined length and intensity (in terms of a heart rate zone). The coach will check whether the patient is indeed correctly following these phases and whether the patient is able to keep the heart rate in the prescribed zone during the core phase. Based on this analysis, the coach will give training-specific advice or suggest the patient to adapt the training program. If the patient is not able to complete exercise sessions according to the prescription, the coach and patient together explore possibilities for an alternative modality or schedule. For example, when the prescribed heart rate zone is not reached during walking, the patient is advised to try biking or running instead. We expect the length of the weekly telephone calls will vary between 10 and 20 minutes, depending on the available training data, encountered problems and training phase.

### Outcome measures

**Figure 1:**
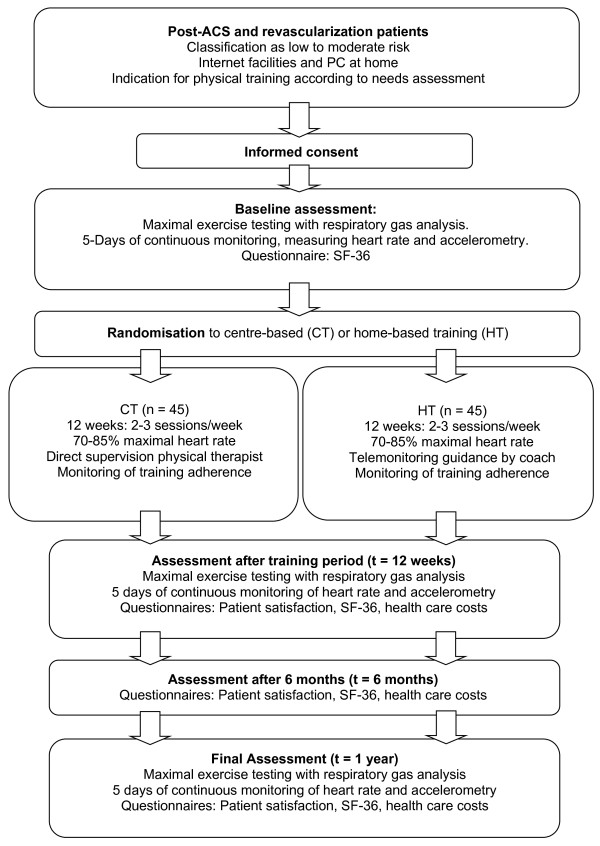
Flowchart of study design.

Main endpoints are physical activity level and physical fitness, assessed at baseline, after 12 weeks and after one year. Secondary endpoints are health related quality of life, patient satisfaction, training adherence and cost-effectiveness. Health related quality of life and patient satisfaction are assessed at baseline, after 12 weeks, after 26 weeks and after one year. Training adherence is assessed in both groups during the 12-week exercise period and costs are assessed after 12 weeks, after 26 weeks and after one year. An overview of the study design is provided in Figure 1.

### Measurements

#### Physical activity level

Physical activity level is assessed by physical activity energy expenditure (PAEE), estimated from accelerometer and heart rate date that are measured during a period of five subsequent days. Participants will wear a compact tri-axial accelerometer (ActiGraph wGT3X + Monitor, ActiGraph) on the hip using an elastic band. The ActiGraph was designed to be worn continuously and previously shown to be a reliable monitor for assessment of daily activity levels [35]. Heart rate is measured with the same heart rate monitor as is used during training sessions at home (Garmin Forerunner 70). It consists of a chest strap and wristwatch receiver with a display providing a continuous reading of the heart rate data. During the assessment periods, patients are instructed to continuously wear both devices during the first night and during day-time of the next five days. Patients are blinded for the accelerometry data. To calculate PAEE, accelerometry data (counts per minute) are time-aligned with heart rate data (beats per minute) and resampled into 20-second epochs. A previously validated branched equation model [36] will be adapted and validated for CR patients and applied to the data to calculate PAEE (Mj per day).

#### Physical fitness

Physical fitness is assessed in both groups by peak oxygen uptake, determined by maximal exercise testing with respiratory gas analysis at the outpatient clinic. This test is performed on a cycle ergometer (Lode Corrival, Groningen), using an individual ramp protocol aiming at a total test duration of 8–12 minutes. Patients are instructed to maintain a pedalling frequency of 70 rounds per minute. A twelve lead ECG is registered continuously. Peak oxygen uptake (peak VO2) is defined as the average value during the last 30 seconds of exercise. In addition, ventilatory thresholds are independently assessed by two physicians who are blinded to the allocation of the patients, using the V-slope method [37]. Assessment of physical fitness is performed at baseline, after three months and after 12 months.

#### Health related quality of life and patient satisfaction

Health related quality of life is assessed by the 36-Item Short Form (SF-36) at baseline, after three months, six months and after 12 months. This questionnaire consists of eight scales ranging from 0–100 and previously showed high reliability with good item-internal consistency, item-discriminant validity and high reliability coefficients in diverse population groups [38]. Patient satisfaction is assessed after three months, using a modified version of the Dutch Consumer Quality-index (CQ-index) for rehabilitation medicine [39]. Both study groups receive identical questionnaires.

#### Training adherence

Training adherence is assessed on a weekly basis during the 12-week rehabilitation period by the physical therapist or coach. Adherence in the CB group is assessed by the number of attended training sessions at the outpatient clinic. For patients in the HT group, the total number of training sessions is assessed via the Garmin Connect web application. This web application also provides insight in the time spent in the prescribed zone (i.e. 70-85% of maximal heart rate) during the training sessions, which is also used to describe adherence to the training program.

#### Cost-effectiveness

In the economic evaluation, the effects of both interventions are compared and related to their difference in costs. Both a cost-effectiveness analysis using the primary outcome measure PAEE as effect measure and a cost-utility analysis using QALYs as outcome measure will be performed. No discounting is applied due to the limited time horizon of one year. The evaluation is performed from a societal perspective, making a distinction between direct (i.e. care-related) and indirect (i.e. other health-related) costs. Direct costs include the costs of rehabilitation such as professional wages (physical therapist, exercise specialist), assessment (exercise testing) and equipment (heart rate monitor), and other healthcare use during the first year of follow up (hospitalizations for recurrent cardiac events, outpatient visits, paramedical visits, general practitioners visits, home care, and informal care). Indirect costs consist of lost productivity costs due to absenteeism from paid and unpaid work. Resource use of the interventions (both HT and CT) is measured prospectively alongside the clinical study as part of the case record form. Other healthcare resource use and absenteeism of paid and unpaid work is collected by means of a questionnaire based on the healthcare consumption, illness and work questionnaire (Tic-P [40]). This questionnaire is filled out by patients after 12 weeks, 6 months and 1 year. For the evaluation of healthcare use, standard prices published in the Dutch costing guideline are used [41]. Costs of absenteeism from paid work are calculated using the friction cost method [41]. In the cost-utility analysis QALYs are calculated from the health utility gain scores obtained with the SF-36 questionnaire at baseline, 12 weeks, 6 months and 1 year [42]. Sensitivity analyses will be carried out on the perspective (healthcare only instead of societal perspective) and method to assess indirect costs (human capital approach instead of friction cost method).

### Sample size analysis

Sample size calculation was performed for the primary endpoint PAEE after 1 year, using data from Bonomi et al. [43]. In this study PAEE in healthy subjects amounted to 4.0 ± 1.2 MJ/day. If the true difference in improvement in PAEE after 1 year between the CT and HT group in this study is 20%, 36 experimental and 36 control subjects need to be studied to be able to test the null hypothesis that the population means are equal (power = 0.8 and alpha = 0.05). Accounting for a 20% loss to follow-up after 1 year (i.e. no assessment at 1 year follow-up), 45 subjects need to be included in both groups.

### Statistical analysis

Data are analysed on an intention-to-treat basis. Multivariate analysis of variance (ANOVA) will be used to assess intra- and intergroup differences as well as their interactions, for PAEE, peak VO2, health related quality of life, patient satisfaction and training adherence. Analyses will be carried out in the statistical programming language R (version 2.13.1).

## Discussion

### Aims of the study

The presented study is designed to evaluate the relative (cost-)effectiveness of home-based exercise training in combination with telemonitoring guidance in low to moderate risk patients entering CR, compared to regular, centre-based exercise training. We hypothesize that early-onset home-based training with online coaching will increase motivation and self-efficacy in CR patients, resulting in improved long-term physical fitness and higher activity levels than after regular centre-based exercise training. The first results of this study are expected in 2014.

### Strengths and limitations

Previous studies indicated that home-based exercise training is equally safe and effective as centre-based exercise training. However, our study distinguishes itself from other studies that have been conducted previously by the use of telemonitoring guidance during home-based exercise training, its long (one year) follow-up period, and by the use of accurate measurement of physical activity energy expenditure in the home environment. To date, the combination of accelerometry and heart rate for the calculation of PAEE has rarely been used in clinical studies. In the last few years, prices for wearable heart rate and accelerometry measurement devices have dropped while their accuracy has increased. With these devices, we can avoid self-reported outcomes based on personal diaries and questionnaires that do not allow for calculation of energy expenditure and have questionable reliability for a combination of different exercise modalities [44]. Our intervention focuses on early activation of patients’ motivation, appealing to their own responsibility and stimulating self-efficacy concerning lifestyle changes. Patients are encouraged to change their lifestyle in their home environment from the onset of the rehabilitation process. This can prevent relapse to an inactive lifestyle, which is often observed after the completion of the typical 12-week supervised centre-based training [11]. In addition, we use telemonitoring for the assessment of training intensity during home-based training. Therefore, coaching by telephone is based on objective training data and is tailored to the individual patient. A study by Aamot et al. [16] showed that home-based high-intensity training, monitored by a HR monitor was equally effective as centre-based high-intensity training with respect to short-term improvement in exercise capacity. Other home-based studies did not monitor training intensity during home-based exercise training [45] or use self-reported perceived exertion rate during training [18,20].

Our study also has some limitations. By design, we are unable to blind participants for allocation. In addition, the personnel supervising the cardiopulmonary exercise tests are not blinded for allocation. During the five days of continuous measurement of physical activity, a bias due to Hawthorne effects is expected. We expect patients to be more active during this period, because they are aware that their daily activity is measured. However, as both groups will experience this Hawthorne effect we expect that this will have no significant effect on the study outcome. The coaching of HT patients is dependent on the Garmin Connect software. Therefore, all patients are required to have internet access from home and basic computer skills to install and use the Garmin Connect software. Although instruction manuals and expert guidance are available, a lack of basic computer skills could occasionally lead to problems at the start of the home-based program. In addition, when a problem with Garmin Connect emerges, or when HT patients forget to upload training data to the Garmin Connect application, effectiveness of the coaching reduced.

### Potential implications for practice

In most countries, only centre-based CR is offered to the vast majority of CR patients. If the results of this study indicate that home-based training with telemonitoring guidance is more effective than centre-based exercise training with respect to long-term physical fitness and physical activity levels, there are compelling reasons to make home-based training equally accessible as centre-based training for low to moderate risk patients, and to stimulate these patients to train at their homes. Also, from a macro-economical point of view it may be beneficial to implement telemonitoring guided home-based CR. Due to underutilization of CR [8], the Dutch Health Inspection has demanded CR centres to increase CR uptake in 2013. However, the current restrictions on healthcare budgets impose great difficulties for Dutch hospitals and CR centres to expand their CR services. A similar trend is visible in other developed countries: despite rising healthcare expenses, uptake of CR is low and budget for expansion of CR is not available [46,47]. Therefore, there is an urgent need for innovative, more cost-effective CR strategies. If home-based training with telemonitoring guidance meets these requirements, it can replace regular centre-based training in CR patients with a low to moderate risk of further events. This will allow a larger number of patients to receive CR without increasing the total costs. This strategy is in line with the 2012 Dutch government budget plans, which include a shift of basic non-complex healthcare services from the hospital towards the patients’ home environment, preferably by large-scale implementation of e-health services.

## Competing interest

The FIT@Home study is executed in collaboration with Philips Research; the heart rate monitors and accelerometers used during the assessment of PAEE and during home-based training are provided by Philips Research.

## Authors’ contributions

NP and HK had the basic idea for this study and were involved in the development of the protocol. HK, NP, MA and JK drafted the manuscript. All authors were involved in the critical revision of the paper for intellectual content and its final approval before submission.

## Pre-publication history

The pre-publication history for this paper can be accessed here:

http://www.biomedcentral.com/1471-2261/13/82/prepub
